# RDW to Platelet Ratio: A Novel Noninvasive Index for Predicting Hepatic Fibrosis and Cirrhosis in Chronic Hepatitis B

**DOI:** 10.1371/journal.pone.0068780

**Published:** 2013-07-17

**Authors:** Baode Chen, Bo Ye, Jian Zhang, Lixiong Ying, Yu Chen

**Affiliations:** 1 Department of Laboratory Medicine, The First Affiliated Hospital of the Medical College, Zhejiang University, Hangzhou, China; 2 Department of Anesthesiology, The First Affiliated Hospital of the Medical College, Zhejiang University, Hangzhou, China; 3 Department of Pathology, The First Affiliated Hospital of the Medical College, Zhejiang University, Hangzhou, China; The University of Hong Kong, Hong Kong

## Abstract

**Objective:**

To develop a simple predictive model for significant fibrosis and cirrhosis in chronic hepatitis B (CHB) using the routine hematological parameters of a complete blood count.

**Methods:**

A total of 458 eligible CHB patients who had undergone a liver biopsy were randomly divided into two cohorts: an estimation group (n = 310) and a validation group (n = 148). Liver histology was assessed according to the Metavir scoring scheme. All common demographics, hematological parameters, HBeAg status, HBV DNA, and liver biochemistry were analyzed.

**Results:**

Based on routinely available clinical parameters (age, sex, HBeAg status, HBV DNA, common hematological parameters of a complete blood cell count), a model for predicting significant fibrosis (Metavir score ≥2) in the estimation group was derived using platelets and red cell distribution width (RDW), and another model for predicting cirrhosis (Metavir score = 4) was derived using platelets, RDW and hemoglobin. A novel index, the RDW to platelet ratio (RPR), was developed to amplify the opposing effects of liver fibrosis on the RDW and platelets. The AUCs of the RPR for predicting significant fibrosis and cirrhosis were 0.825 and 0.884, respectively, which is superior to the AAR, FIB-4 and APRI in the estimation group. Compared with the two derived models, the RPR has a comparable predictive power for significant fibrosis and cirrhosis. Using optimized cutoffs (0.10 and 0.16), the RPR accurately predicted 63.1% of cases with significant fibrosis and 73.7% of cases with cirrhosis and accurately excluded 85.5% of the cases with mild fibrosis and 93.0% of the cases with no cirrhosis.

**Conclusion:**

The RPR, a routinely available, inexpensive and easily calculated index, can predict significant fibrosis and cirrhosis in CHB patients with relatively high accuracy. The application of this index may reduce the need for liver biopsy in CHB patients.

## Introduction

Liver fibrosis and cirrhosis are major causes of morbidity and mortality in chronic hepatitis B (CHB) patients. Although antiviral therapy has greatly reduced the risk of fibrosis and cirrhosis, some patients may eventually develop advanced fibrosis and cirrhosis. Knowledge of the stages of liver fibrosis is essential in patients with viral hepatitis B to assess the progression and prognosis of the disease, particularly when determining whether to use antiviral treatment [1], [2]. Currently, liver biopsy remains the gold standard for assessing the histological outcomes of liver disease [3]. However, this procedure is costly and carries a small risk of complications due to sampling error and invasiveness [4]. Thus, non-invasive, economical and simple methods to assess the severity of hepatic fibrosis are considered to be attractive.

Several non-invasive methods using laboratory tests, scores and indices to predict hepatic fibrosis have been proposed over the past decade, such as the FibroTest and Fibroindex, AST/ALT ratio, APRI and FIB-4 [5], [6], [7], [8], [9]. However, these methods were based on patients with hepatitis C and might produce conflicting results in the prediction of liver fibrosis among CHB patients. Recently, several models based on CHB patients have been reported, but they are somewhat difficult to use in clinical practice because these models utilize less common biochemical markers or require the use of a special computer program to perform the calculations [10], [11]. Moreover, the accuracy and reliability of these assays are not very satisfactory in predicting liver fibrosis. Thus, additional studies are necessary to identify a simple, accurate and routinely available method.

The complete blood count (CBC) is one of the most frequently ordered laboratory tests in clinical practice. Standard CBC tests include white blood cell (WBC), red blood cell (RBC) and platelet counts as well as their morphological indices. Various studies have evaluated the performance of these hematological CBC parameters to predict disease severity and mortality risk. For example, the circulating platelet count has been proposed as a biomarker of liver fibrosis and cirrhosis [12]. An elevated red cell distribution width (RDW) has been reported to be associated with mortality and other severe adverse outcomes in cardiac, renal and infectious diseases, even in the general population [13], [14], [15], [16]. Other studies have found an association between low hemoglobin (Hb) concentrations and mortality [17]. Despite these correlations, few studies have evaluated the association between these parameters and the histological outcomes of liver disease in patients with CHB. Given the relative ease and low cost of measuring these indices and their close association with disease progression and prognosis, we attempted to develop a simple clinical approach using these measurements to predict the liver fibrosis stages in patients with chronic hepatitis B.

## Materials and Methods

### Patients and Laboratory Assessment

Our cohort comprised 603 patients with CHB who underwent liver biopsy to assess the degree of liver fibrosis and necroinflammation at the First Affiliated Hospital of the School of Medicine from Feb 2008 to Jan 2012. CHB is diagnosed when serum hepatitis B surface antigen (HBsAg) is positive for more than 6 months and when persistent or intermittent elevations in ALT/AST levels and symptoms or signs of hepatitis or histological changes are present [18]. For all subjects, demographic, clinical and laboratory data were collected. All laboratory variables were obtained within 7 days of the liver biopsy.

In this study, the exclusion criteria included co-infection with HCV, hepatitis D virus, hepatitis G virus and/or human immunodeficiency virus, other autoimmune liver disease, hepatocellular carcinoma, liver transplantation and metabolic liver disease. Patients who received antiviral or anticoagulant treatment 6 months before admission were also excluded. The final subjects were randomly divided according to a computer-generated randomization schedule into an estimation group (approximately two thirds of the subjects) and a validation group (approximately one third of the subjects). All subjects gave written informed consent for the liver biopsy. The Ethics Committee of the First Affiliated Hospital of the Medical School of Zhejiang University, Hangzhou, China, approved the consent procedure and the study.

### Histological Assessment of the Liver

Liver biopsy was performed using a fully automatic biopsy needle (18G BioPince™, InterV-MDTech, Gainesville, Florida) under ultrasound guidance. A minimum of 1.5 cm of liver tissue with at least five portal tracts was required for diagnosis. The specimens were obtained using H&E and Masson’s trichrome stains on formalin-fixed paraffin-embedded liver tissue. All histological specimens from the eligible patients were reviewed by a pathologist, who was blind to the patient characteristics, according to the Metavir scoring scheme [19]. When the diagnostic results were not consistent with the previous results, another pathologist was invited to make a further assessment. Fibrosis was scored on a 5-point scale: F0 (no fibrosis), F1 (portal fibrosis without septa), F2 (portal fibrosis with rare septa), F3 (portal fibrosis with many septa), and F4 (cirrhosis). Significant fibrosis was considered to be a fibrosis score of 2 or more (F2, F3 and F4), advanced fibrosis was defined as a fibrosis score of 3 or more(F3 and F4) and cirrhosis was defined as a fibrosis score of 4 (F4).

### Non-invasive Prediction Methods and Calculation Formulae

The non-invasive prediction methods used in our study included the AST-to-ALT ratio (AAR), AST-to-platelet ratio index (APRI) and FIB-4. The calculation formulae were as follows: 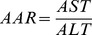 [20]; 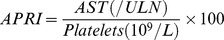, where ULN is the upper limit of normal of the AST level [7]; 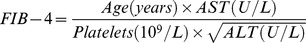 [21].

### Statistical Analyses

The data analysis was performed using SPSS version 15.0 software (SPSS Inc., Chicago, IL, United States). All continuous variables were analyzed for the normality of distribution after logarithmic transformation. Differences between the estimation and validation groups were tested using Student's *t*-test for continuous variables and the Χ^2^ -test for categorical variables. Correlations were evaluated by the Spearman correlation coefficient. Hematological parameters were presented as the quartile ranks for the univariate and multivariate analyses to select independent predictors. Binary logistic regression analyses were used to develop the predictive models of significant fibrosis and cirrhosis. Probabilities generated from the predictive models were recorded and used as new input variables for the receiver operating characteristic (ROC) curve analysis. The accuracy of the models and RDW-platelet ratio (RPR) was evaluated by constructing ROC curves. Differences between the AUCs were tested using the z-test. The probability cut-off points for the optimal combination of sensitivity and specificity were determined by the Youden index. The predictive models and RPR were validated by the standard diagnostic analysis of sensitivity, specificity and positive and negative predictive values. All *p*-values were 2-sided, and values <0.05 were considered statistically significant.

## Results

### Study Population

A total of 603 patients with CHB who had undergone a percutaneous liver biopsy were enrolled in the study. Overall, 30 patients were excluded due to insufficient data, and 115 were excluded because of hepatocellular carcinoma, liver transplants, other liver diseases and medicine usage. The final study cohort, consisting of 458 subjects, was divided into two groups: an estimation group containing 310 patients and a validation group containing 148 patients. The demographic and clinical characteristics of the subjects are shown in Table 1. All variables were comparable between the estimation and validation groups.

**Table 1:** Baseline characteristics of the subjects.

Variables	Estimation group (N = 310)	Validation group (N = 148)	*p-*value
Demographic			
Age (years)	42.1±11.4	40.3±11.2	0.105
Male	183 (59.0%)	97 (65.5%)	0.181
Hepatitis virus			
HBeAg positivity	205 (66.1%)	96 (64.8%)	0.790
Detectable HBV DNA	224 (72.3%)	102 (68.9%)	0.461
Histological (Metavir scores)			
0–1	175 (56.5%)	83 (56.1%)	0.940
2	57 (18.4%)	25 (16.9%)	0.696
3	40 (12.9%)	21 (14.2%)	0.705
4	38 (12.3%)	19 (12.8%)	0.860
Hematological Parameters			
WBC count (10^9^/L)	5.89±3.21	5.83±2.57	0.847
RBC count (10^12^/L)	4.98±1.41	4.84±1.39	0.342
Hemoglobin (g/L)	135.35±24.58	137.31±22.65	0.402
RDW (%)	14.22±2.83	13.77±1.74	0.069
MCV (fL)	89.98±7.61	88.72±9.61	0.132
Platelet count (10^9^/L)	150.37±51.96	157.09±55.78	0.207
PDW (%)	15.23±2.89	15.08±3.15	0.627
MPV (fL)	11.48±1.57	11.46±1.54	0.888
Liver function markers			
AST (U/L)	91.91±133.70	82.63±128.57	0.357
ALT (U/L)	103.16±152.48	97.52±142.64	0.692
ALP (U/L)	98.23±136.92	81.18±110.23	0.586
Total bilirubin (mmol/L)	36.22±63.53	29.10±41.11	0.182

Data are expressed as the means±SD or N (%).

AST: aspartate aminotransferase; ALT: alanine aminotransferase; ALP: alkaline phosphatase. WBC: white blood cell; RBC: red blood cell; RDW: red cell distribution width; MCV: mean corpuscular volume; PDW: platelet distribution width; MPV: mean platelet volume.

### Predictors and Regression Models

In the univariate analysis, an increased odds ratio (OR) of significant fibrosis was associated with age (OR = 1.04, *p*<0.001) and the fourth (vs. first) quartiles (Qs) of hemoglobin (OR = 1.79, *p*<0.001), RDW (OR  = 2.18, *p*<0.001) and platelet count (OR = 2.75, *p*<0.001). Similarly, an increased OR of cirrhosis was found for hemoglobin (OR = 3.43, *p*<0.001), RDW (OR  = 3.85, *p*<0.001) and platelet count (OR = 3.74, *p*<0.001). These significant variables were selected for a multivariate analysis. In the multivariate analysis, only RDW and platelets remained as independent predictors of significant fibrosis, and hemoglobin, RDW and platelets were retained as independent predictors of cirrhosis (Table 2). Finally, based on these independent predictors, two regression models were derived to predict significant fibrosis and cirrhosis, respectively. The models are as follows:

**Table 2:** Univariate and multivariate analyses of the relationships between the fourth (vs. first) quartile hematological parameters and liver fibrosis and cirrhosis in the estimation patients.

Variables	Significant fibrosis (F2–F4)						Cirrhosis(F4)			
	Univariate	*p-*value	Multivariate		*p-*value		Univariate	*p-*value	Multivariate	*p-*value
Age (per decade)	1.04 (1.02,1.07)	<0.001		1.01 (0.98,1.05)		0.525	1.03 (1.00,1.06)	0.107		
Gender (male/female)	0.99 (0.63,1.57)	0.943					0.85 (0.42,1.73)	0.367		
HBeAg (positive/negative)	0.58 (0.31,1.01)	0.086		0.78 (0.35,1.76)		0.555	0.74 (0.31,1.76)	0.375		
Detectable HBV DNA (yes/no)	0.65 (0.32,1.32)	0.186					0.63 (0.23,1.80)	0.346		
WBC count (Q4 vs. Q1)	0.95 (0.78,1.16)	0.227					1.15 (0.82,1.51)	0.106		
RBC count (Q4 vs. Q1)	1.01 (0.97,1.03)	0.667					1.02 (0.99,1.06)	0.234		
Hemoglobin (Q4 vs. Q1)	1.79 (1.44,2.23)	<0.001		1.01 (0.68, 1.51)		0.953	3.43 (2.14,5.49)	<0.001	2.15 (1.27, 3.66)	0.002
RDW (Q4 vs. Q1)	2.18 (1.73,2.75)	<0.001		1.80 (1.20, 2.69)		0.004	3.85 (2.38,6.23)	<0.001	2.06 (1.21, 3.51)	<0.001
MCV (Q4 vs. Q1)	1.00 (0.82,1.23)	0.988					1.03 (0.82,1.30)	0.520		
Platelet count (Q4 vs. Q1)	2.75 (2.13,3.55)	<0.001		2.65 (1.86, 3.78)		<0.001	3.74 (2.29,6.10)	<0.001	2.55 (1.59, 4.09)	<0.001
PDW (Q4 vs. Q1)	0.97 (0.81,1.23)	0.967					1.17 (0.91,1.51)	0.930		
MPV (Q4 vs. Q1)	0.99 (0.80,1.22)	0.928					0.98 (0.76,1.27)	0.103		

Data in the univariate and multivariate analyses are expressed as odds ratios (95% CI).

WBC: white blood cell; RBC: red blood cell; RDW: red cell distribution width; MCV: mean corpuscular volume; PDW: platelet distribution width; MPV: mean platelet volume.

Regression model for the prediction of significant fibrosis:
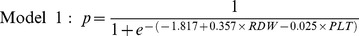



Regression model for the prediction of cirrhosis:
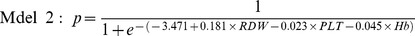



### A Novel Index Consisted of RDW and Platelets for Predicting Liver Fibrosis

**Figure 1:**
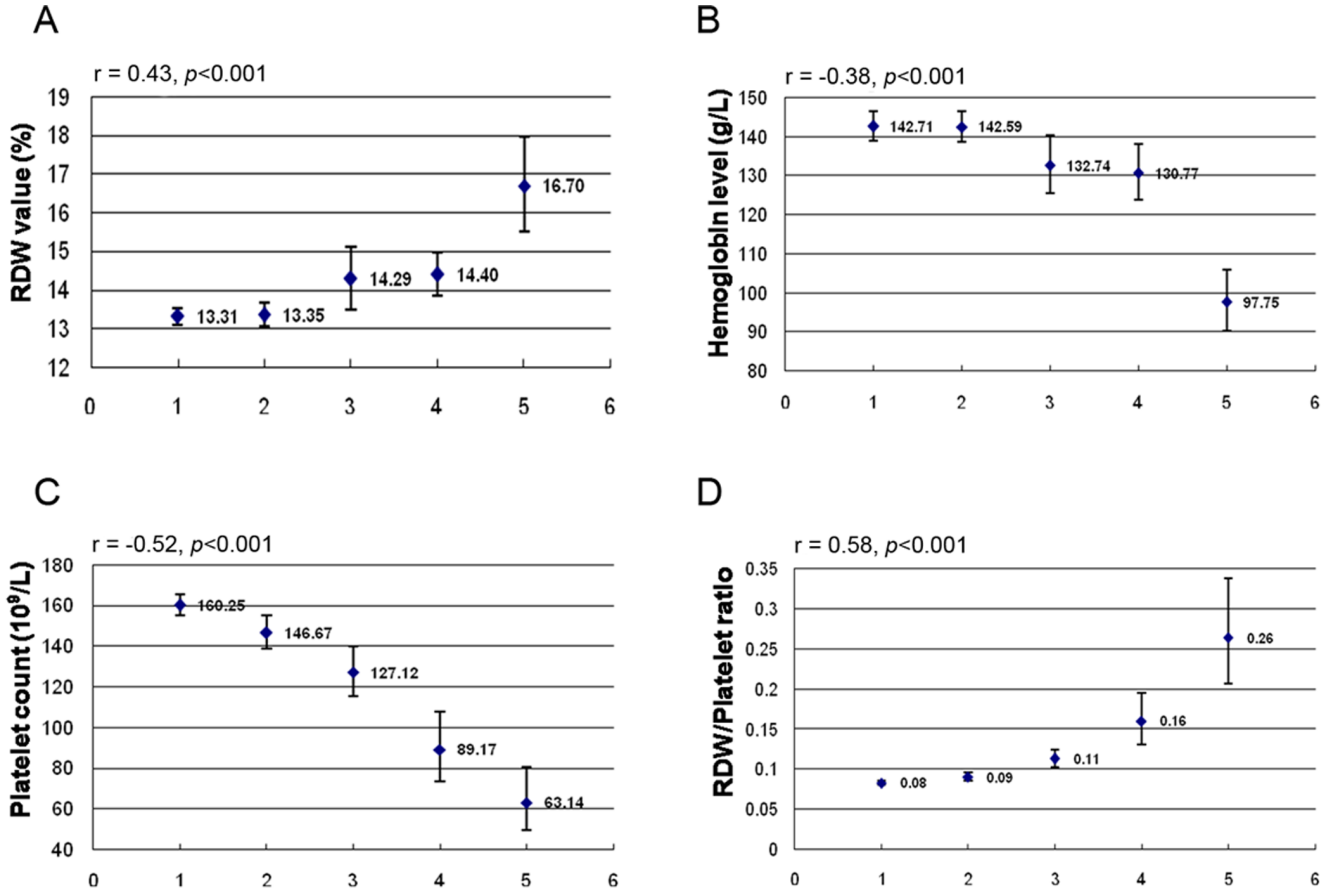
Geometric means and 95% CIs of RDW (A), hemoglobin (B), platelet count (C) and the RDW/platelet ratio (D) in the five subgroups (F0, F1, F2, F3 and F4) classified by fibrosis stage (Metavir scores). r is the correlation coefficient of the fibrosis stages with RDW, hemoglobin, platelet count and the RDW/platelet ratio.



The associations of hemoglobin, RDW and platelets with histological outcomes were further analyzed. Among these significant independent predictors, RDW was positively correlated with significant fibrosis and cirrhosis, whereas platelets and hemoglobin were negatively correlated with significant fibrosis and cirrhosis. Figure 1 shows that the severity of liver fibrosis was correlated significantly with a gradual increase in the RDW (Fig. 1 A) and with a decrease in hemoglobin and platelets (Fig. 1 B, C). Compared with hemoglobin (*r* = −0.38), a decreased platelet count (*r* = −0.52) was more closely associated with the stage of fibrosis. Thus, RDW and platelets were the most powerful risk predictive factors of liver fibrosis. Because an overlap existed in the different stages of fibrosis, we devised the RDW to platelet ratio (RPR) to amplify the difference in the RDW and platelets among patients with different fibrosis stages.

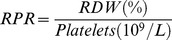



Consequently, the RPR was significantly related to the stage of fibrosis, with a higher correlation coefficient than the RDW or platelets alone (*r* = 0.58) (Fig. 1 D).

### Accuracy of the RPR, Model 1, Model 2, AAR, FIB-4 and APRI in Predicting Liver Fibrosis and Cirrhosis in the Estimation Group

**Figure 2:**
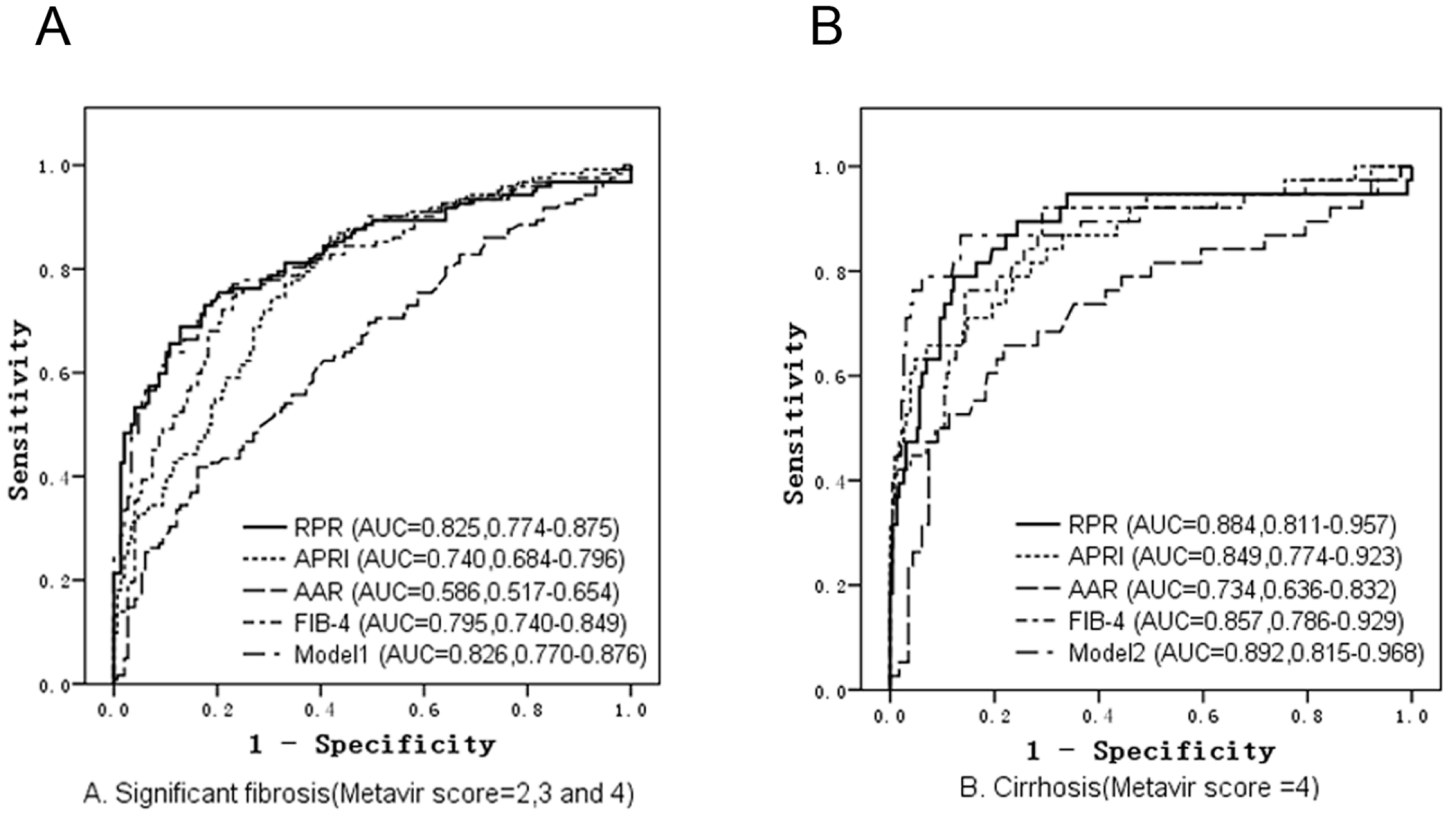
Receiver operating characteristic (ROC) curves of RPR, APRI, AAR, FIB-4 and the models in the prediction of significant fibrosis (A) and cirrhosis (B) for the estimation patients.

An ROC curve analysis was applied to estimate the predictive values of the RPR, Model 1 and Model 2 in the estimation group. Meanwhile, the RPR was compared to three pre-existing non-invasive indexes including the AAR, APRI and FIB-4. The AUCs of all six models for predicting significant fibrosis and cirrhosis in the estimation group are shown in Figures 2 A and B. The AUCs of the RPR, APRI, AAR, FIB-4 and Model 1 were 0.825, 0.740, 0.586, 0.795 and 0.826, respectively, in the prediction of significant fibrosis. The AUCs of the RPR, APRI, AAR, FIB-4 and Model 2 were 0.884, 0.849, 0.734, 0.857 and 0.892, respectively, in the prediction of cirrhosis. The RPR exhibited a significantly higher AUC in the prediction of significant fibrosis compared to the APRI and AAR (*p*<0.05) and in the prediction of cirrhosis compared to the AAR (*p*<0.05). No significant difference was observed between the RPR, FIB-4 and Model 1 in the prediction of significant fibrosis (*p*>0.05) or between the RPR, APRI, FIB-4 and Model 2 in the prediction of cirrhosis (*p*>0.05).

### Validation and Comparison of RPR, Model 1, Model 2, AAR, FIB-4 and APRI in Predicting Liver Fibrosis and Cirrhosis in the Validation Patients

The diagnostic performance of the models, RPR, AAR, FIB-4 and APRI was assessed using data from the validation group. The sensitivity, specificity, negative predictive value and positive predictive value of all six models, using the various cutoffs suggested for each model, are shown in Table 3. Among the 83 patients without significant fibrosis, 71 (85.5%) had an RPR score less than the optimal cutoff of 0.1, and only 55 (66.3%) had an APRI score less than the cutoff of 0.5, suggesting that the patients who did not have significant fibrosis were classified correctly and that liver biopsies could thus be avoided. Among the 108 patients without advanced fibrosis, 79 (73.1%) had an FIB-4 score less than the cutoff of 1.45. Among the 40 patients with advanced fibrosis, 10 (25.0%) had an FIB-4 score greater than the cutoff of 3.25. For the 65 patients with significant fibrosis, 41 (63.1%) had an RPR score greater than the optimal cutoff of 0.1, and 49 (75.4%) had an APRI score greater than the cutoff of 1.5. Similarly, among the 129 patients without cirrhosis, 120 (93.0%) had an RPR score less than the optimal cutoff of 0.16, 121 (93.8%) had an APRI score less than the cutoff of 2.0, and 124 (96.1%) had an AAR score less than the cutoff of 1.0. For the 19 patients with cirrhosis, 14 (73.7%) had an RPR score greater than the optimal cutoff of 0.16, 12 (61.4%) had an APRI score greater than the cutoff of 2.0, and 13 (68.4%) had an AAR score greater than the cutoff of 1.0, thus suggesting that these patients should have been considered for more stringent monitoring and management.

**Table 3:** Validation and comparison of RPR, the models, AAR, FIB-4 and APRI for the prediction of fibrosis stages in the validation patients.

Noninvasive Test	Cutoffs	Patients classified N (%)	Diagnostic accuracy	Metavir score		
				F2–4	F3–4	F4
RPR	0.10	53 (35.8)	Sen (%)	63.1		
			Spe (%)	85.5		
			PPV (%)	77.4		
			NPV (%)	74.7		
	0.16	23 (15.5)	Sen (%)			73.7
			Spe (%)			93.0
			PPV (%)			60.8
			NPV (%)			96.0
Model 1	0.44	56 (37.8)	Sen (%)	69.2		
			Spe (%)	86.7		
			PPV(%)	80.4		
			NPV (%)	78.3		
Model 2	0.30	22 (14.9)	Sen (%)			68.4
			Spe (%)			93.0
			PPV (%)			59.1
			NPV (%)			95.2
AAR	1.0	18 (12.2)	Sen (%)			68.4
			Spe (%)			96.1
			PPV (%)			72.2
			NPV (%)			95.4
FIB-4	1.45	56 (37.8)	Sen (%)		67.8	
			Spe (%)		72.7	
			PPV (%)		48.2	
			NPV (%)		85.9	
	3.25	15 (10.1)	Sen (%)		25.1	
			Spe (%)		95.7	
			PPV (%)		66.7	
			NPV (%)		77.4	
APRI	0.5	77 (52.0)	Sen (%)	75.4		
			Spe (%)	66.3		
			PPV (%)	63.6		
			NPV (%)	77.5		
	1.5	28 (21.9)	Sen (%)	33.8		
			Spe (%)	92.8		
			PPV (%)	78.6		
			NPV (%)	64.2		
	1.0	55 (37.2)	Sen (%)			85.0
			Spe (%)			69.7
			PPV (%)			29.1
			NPV (%)			96.7
	2.0	20 (13.5)	Sen (%)			63.2
			Spe (%)			93.8
			PPV (%)			60.0
			NPV (%)			94.5

The cutoffs described in the original reports were used for all calculations. For AAR, Metavir stage F4 in our study was compared with the presence of cirrhosis in the Giannini study [20]; for FIB-4, Metavir stages F3–F4 in our study were compared with the presence of advanced fibrosis (Ishak stages F4–F6) in the Richard K study [21]; and for APRI, Metavir stages F2–F4 in our study were compared with the presence of significant fibrosis (Ishak stages F3–F6), and F4 was compared with the presence of cirrhosis (Ishak stages F5–F6) in the Wai study [7].

RPR: RDW-to-platelet ratio; AAR: AST-to-ALT ratio; APRI: AST-to-platelet ratio index; Sen: sensitivity; Spe: specificity; PPV: positive predictive value; NPV: negative predictive value.

## Discussion

The aim of this study was to develop a simple, inexpensive and routine approach to predict significant fibrosis and cirrhosis using easily available hematological CBC parameters. Although the search for new biomarkers is quite active, information on the stages of liver fibrosis in patients with chronic hepatitis B is available from routine hematologic tests, such as the complete blood count. Anemia (low hemoglobin) has been long known to be associated with an increased risk of mortality, and hemolytic anemia is commonly present in chronic liver disease, particularly cirrhosis [22], [23]. Thrombocytopenia has also been a well-known predictor of severe liver fibrosis. The platelet count has been used in the most predictive models for liver fibrosis and cirrhosis. The RDW, an indicator of the variability of the circulating RBC size, is often used to diagnose different types of anemia. Recent studies have reported that a higher RDW is associated with a higher mortality risk in various patient populations. A prospective study by Kushang V. Patel l et al showed that the RDW was a strong predictor of mortality in middle-aged and older adults [14]. Another study by Lou et al reported that increased RDW values were associated with disease severity in patients with hepatitis B [24]. Consistent with these correlations, we found that 3 hematologic CBC parameters–hemoglobin, RDW and platelets–were independent predictors of the liver fibrosis stage in patients with chronic hepatic B. In addition, our findings indicated that the other parameters were not predictive risk factors for liver fibrosis. In contrast, a previous study concluded that WBCs and their subtypes were associated with histological severity in patients with nonalcoholic fatty liver disease [25]. Two other retrospective studies revealed that the mean platelet volume (MPV) was increased in chronic hepatitis C patients with advanced fibrosis [26], [27]. These inconsistencies could result from the different study populations, which had liver disease caused by different pathogenic factors. Although increasing evidence highlights the prognostic value of individual CBC parameters [28], [29], to our knowledge, our study is the first to report the relationship between RDW and the stage of liver fibrosis. Moreover, few studies have combined the RDW and platelet count, the two strongest predictors, to identify CHB patients with significant fibrosis or cirrhosis. Our study addressed this gap and found that the RDW and the RDW to platelet ratio in particular could predict the risk of significant liver fibrosis and cirrhosis.

The RPR and regression models based on CBC parameters all exhibited excellent performance in the prediction of significant fibrosis and cirrhosis. The RPR index obtained an AUC of 0.825 in the prediction of significant fibrosis. The AUC improved to 0.884 in the prediction of cirrhosis. The sensitivity and specificity of the RPR (63.1% and 85.5.0%, respectively, for significant fibrosis and 73.7% and 93.0%, respectively, for cirrhosis) were both high at the optimal cutoffs. The two regression models, which required more variables and complicated calculations, obtained higher AUCs than the RPR but did not significantly improve the predictive ability of significant fibrosis and cirrhosis. Compared with the APRI and FIB-4, the RPR is a more powerful predictive index of significant fibrosis but is equally powerful in the prediction of cirrhosis. The AAR has no advantage in predicting significant fibrosis and cirrhosis. The APRI was initially created based on patients with chronic hepatitis C. Although the APRI is a promising tool for predicting hepatic fibrosis and includes only 2 inexpensive laboratory tests that are performed routinely in all patients, a meta-analysis demonstrated that the APRI was not an appropriate choice to identify hepatitis B-related fibrosis in CHB patients [30]. Moreover, the appropriate definition of the upper limit of normal (ULN) of the AST level remains uncertain because each laboratory uses a different ULN value. Other models, such as the FIB-4, FibroTest-ActiTest and FibroScan (all based on chronic hepatitis C patients), were applicable to patients with CHB in some investigations but were limited by the requirement of complicated calculations or expensive instruments [31], [32], [33], [34]. In addition, two scoring systems, constructed by Hui AY and Zeng MD, respectively, were based on CHB patients but were applied to special populations: patients with an HBV-DNA level >10^5^ copies/ml and patients who were HBeAg positive, respectively [10], [11]. Most recently, Wai-Kay Seto and colleagues introduced a new model, the PAPAS index, which still requires cumbersome calculations [35]. Altogether, compared with the current predictive models, the RPR requires only 2 common CBC parameters and is the simplest, cheapest and most easily calculated non-invasive method with a relatively high accuracy.

The development of liver fibrosis is considered to be a complex trait. The role of platelets in the progression of fibrosis is not well understood. Recent findings have revealed a potentially beneficial role of platelets, in which they have been found to alleviate liver fibrosis through the decreased expression of the principal profibrogenic cytokine TGF-β and the increased expression of matrix metalloproteinases [36]. Nevertheless, a negative correlation exists between liver fibrosis progression and platelets. Platelets contribute to the inflammatory reaction after liver injury [37]. Platelets thus appear to have a dual role in liver fibrogenesis and regeneration [38], [39]. Additionally, the mechanism underlying the association between the RDW and the stage of liver fibrosis in CHB patients is unclear. A prospective, multicenter study suggested that an elevated RDW might indicate inflammatory stress and impaired iron mobilization [40]. Indeed, inflammation and iron overload play key roles in mediating the processes associated with hepatic fibrosis [41]. Richard G Ruddell et al demonstrated a role for ferritin, an indicator of body iron stores, in regulating the expression of proinflammatory cytokines associated with hepatic fibrogenesis [42]. Furthermore, inflammatory cytokines may increase the heterogeneity of erythrocyte maturation and impairment, characterized by an elevated RDW.

The present study had several limitations. The patients were retrospectively enrolled, and not all were treatment-naive CHB patients. However, CHB patients who had recently received antiviral and anticoagulant treatments were excluded to avoid drug interference in the measurement of hematological parameters. In addition, laboratory results were collected within one week of the liver biopsy, which could lead to a measurement bias; thus, we used the set of results that was recorded closest to the time of the biopsy. In addition, the HBV genotypes of the patients were not assessed due to the absence of available data. Although genotype C HBV has been shown to be related to more active liver disease than genotype B HBV in Asian patients [43], the determination of the HBV genotype is not a routine clinical test. Finally, this study involved a single center, such that the small proportion of patients with significant fibrosis and/or cirrhosis may have posed a selection bias in this study. Therefore, the predictive performance of the RPR should be further confirmed in multi-center, prospectively designed studies.

In conclusion, the present study provided important insights into the progression and prognosis of chronic hepatitis B using a complete blood cell count. Two common hematological parameters, the RDW and platelet count, provided the greatest predictive value of liver fibrosis. The RPR, an inexpensive and easily calculated index, can predict significant fibrosis and cirrhosis in CHB patients with relatively high accuracy, potentially reducing unnecessary liver biopsies. Further studies are needed to validate this index and compare it with other non-invasive methods of liver fibrosis in CHB patients.
